# Expression of parvin-*β* is a prognostic factor for patients with urothelial cell carcinoma of the upper urinary tract

**DOI:** 10.1038/sj.bjc.6605835

**Published:** 2010-08-24

**Authors:** C-F Wu, K-F Ng, C-S Chen, P-L Chang, C-K Chuang, W-H Weng, S-K Liao, S-T Pang

**Affiliations:** 1 Department of Surgery, Division of Urology, Chia-Yi Chang Gung Memorial Hospital, Chia-Yi, Taiwan; 2 Graduate Institute of Clinical Medical Sciences, Chang Gung University, Kwei-shan, Taoyuan, Taiwan; 3 Department of Pathology, Lin-Kou Chang Gung Memorial Hospital, Kwei-shan, Taoyuan, Taiwan; 4 Department of Surgery, Division of Urology, Lin-Kou Chang Gung Memorial Hospital, No. 5, Fushing Road, Kwei-Shan, Taoyuan, Taiwan; 5 Chang Gung Bioinformatics Center, Lin-Kou Chang Gung Memorial Hospital, Kwei-shan, Taoyuan, Taiwan; 6 Department of Chemical Engineering and Biotechnology, Graduate Institute of Biotechnology, National Taipei University of Technology, Taipei, Taiwan; 7 Cancer Immunotherapy Program, Taipei Medical University Hospital, Taipei, Taiwan

**Keywords:** parvin-*β*, urothelial cell carcinoma, upper urinary tract, prognostic factor

## Abstract

**Background:**

Parvin-*β* (ParvB), a potential tumour suppressor gene, is a focal adhesion protein. We evaluated the role of ParvB in the upper urinary tract urothelial cell carcinoma (UUT-UC).

**Methods:**

ParvB mRNA and proteins levels in UUT-UC tissue were investigated by quantitative real-time polymerase chain reaction and western blot analysis, respectively. In addition, the expression of ParvB in tissues from patients with UUT-UC at different stages was evaluated by immunohistochemistry. Furthermore, biological functions of ParvB in urothelial cancer cells were investigated using a doxycycline-inducible overexpression system and siRNA.

**Results:**

Western blot and mRNA analysis showed downregulation of ParvB expression in frozen UUT-UC tissue. Immunohistochemistry revealed high staining intensity of ParvB in normal urothelium, which decreased markedly at advanced stages of UUT-UC (*P*=0.0000). Moreover, ParvB was an independent prognostic indicator for disease-specific survival of patients with UUT-UC. Functional assays indicated that overexpression of ParvB in an urothelial cancer cell line resulted in decreased cell growth rate and ability to migrate. In contrast, knockdown of ParvB expression increased cell migration ability.

**Conclusions:**

Downregulation of ParvB expression significantly increased urothelial cancer cell growth and migration. Downexpression of ParvB level in UUT-UC correlated with tumour stage, and was an independent unfavourable prognostic factor for disease-specific survival of patients with UUT-UC.

Urothelium cell carcinoma of the upper urinary tract (UUT-UC), which includes the renal pelvis, calyx and ureter, is relatively uncommon and accounts for ∼5% of all urothelial tumours and ∼10% of all renal tumours (Krogh *et al*, 1991; Guinan *et al*, 1992). However, the incidence of patients with UUT-UC who initially present with local advancement and metastatic tumour is approximately ∼45% (Charbit *et al*, 1991; Racioppi *et al*, 1997; Kang *et al*, 2003). Tumour stage and grade have been documented as major prognostic factors in patients with UUT-UC (Cozad *et al*, 1995; Hall *et al*, 1998; Kirkali and Tuzel, 2003; Li *et al*, 2008). Furthermore, even after undergoing radical surgery followed by adjuvant therapy, UUT-UC patients with a high pT stage show poor clinical outcomes owing to high local tumour recurrence and distant metastases (Lerner *et al*, 1996; Maulard-Durdux *et al*, 1996; Bamias *et al*, 2004; Czito *et al*, 2004; Kwak *et al*, 2006). On the basis of the above clinical findings, studies seeking to understand UUT-UC at the cellular level are necessary for the development of novel therapeutic approaches and improved treatment strategies.

Cell–extracellular matrix (ECM) adhesion is crucial in various biological processes, including cell migration, proliferation, gene expression and cell survival (Hynes, 2002; Guo and Giancotti, 2004; Lee and Juliano, 2004). Proteins that facilitate cell–ECM adhesion include integrin-linked kinase (ILK), talin, PINCH, paxillin, *α*-parvin (ParvA), *β*-parvin (ParvB) and focal adhesion kinase. These proteins are critically involved in the regulation of cell survival and morphology (Tu *et al*, 1999, 2001; Nikolopoulos and Turner, 2001; Olski *et al*, 2001; Wu and Dedhar, 2001; Yamaji *et al*, 2001). Evidence suggests that a PINCH–ILK–parvins complex is a key component that mediates cell–ECM adhesion (Legate *et al*, 2006). Proteins belonging to the parvin family, including ParA, ParB and *γ*-parvin (ParvG), in mammals are coded by three different genes. These parvins possess identical domain architecture and share a high level of sequence similarity (Korenbaum *et al*, 2001; Sepulveda and Wu, 2006). Although ParvA and ParvB are widely expressed and show overlapping expression in various tissues, ParvG expression is restricted to the haematopoietic system (Korenbaum *et al*, 2001). Parvin-*β* maps to chromosome 22q13.31 (Korenbaum *et al*, 2001). Castells *et al* (2000) showed that a deletion on chromosome 22q13 is involved in breast and colorectal cancer and suggested the existence of tumour suppressor genes within this region (Castells *et al*, 2000). Moreover, Mongroo *et al* (2004) showed that ParvB inhibits ILK-mediated signalling and that ParvB is downregulated in advanced breast cancer. In our previous studies, we also found that ParvB is downregulated in transitional cell carcinoma by utilising a cDNA microarray technique (unpublished data). Here, we further characterise the biological roles of ParvB in UC and evaluate its potential biomarker role in UUT-UC.

## Materials and methods

### Patients and tumour specimens

From January 1998 to December 2008, patients with UUT-UC treated at Lin-Kou and Chia-Yi Chang Gung Memorial Hospital were reviewed retrospectively. Immunohistochemical staining were performed using 129 paraffin-embedded UUT-UC specimens retrieved from the Department of Pathology; disease-free survival analyses were conducted on the basis of clinical chart reviews. Nineteen pairs of normal and urothelial tumour specimens obtained from the Chung Gung Memorial Hospital Tissue Bank were used for quantitative real-time polymerase chain reaction (PCR). Seven patients had non-muscle invasive tumours (Ta, T1) and 12 had muscle invasive tumours (T2, T3 and T4). Tumour grade was evaluated using 1998 World Health Organization grading system (Epstein *et al*, 1998), whereas tumour stage was determined using 2002 AJCC TNM classifications (Greene *et al*, 2002).

### RNA extraction and quantitative RT–PCR

Total RNA extraction from tumour tissue, normal urothelium and cell lines was performed using Trizol (Invitrogen, Carlsbad, CA, USA) according to the manufacturer's instructions. Total RNA was quantified and qualified by spectrophotometry (NanoDrop Technology Inc., Wilmington, DE, USA). RNA samples treated with RQ1 RNase-Free DNase I (Promega, Madison, WI, USA) were reverse transcribed with SuperScript (Invitrogen) using oligo-dT primers. Quantitative real-time PCR (Q-PCR) reactions were performed in a total volume of 20 *μ*l using SYBR GreenSuperMix (BioRad, Hercules, CA, USA) and a BioRad iCycler iQ Real-Time Detection System according to the manufacturer's instructions. Primers with the following sequences were used: ParvB – forward, 5′-AATGTGGCTGAGGTGACACA-3′ and reverse, 5′-TCTTCCCGTGAATTGAGTCC-3′ GAPDH – forward, 5′-GAAGGTGAAGGTCGGAGTC-3′ and reverse, 5′-GAAGATGGTGATGGGATTTC-3′. Quantitative real-time PCR cycle conditions were 3 min at 95°C, 15 s at 95°C and 45 s at 60°C for 45 cycles. Polymerase chain reaction reactions were performed in triplicate and relative expression levels of ParvB in normal and tumour tissues were calculated by normalising to GAPDH expression levels by using the comparative *C*_T_ method. *C*_T_ represents the cycle numbers at which the amplification reaches a threshold level that is chosen to lie within the exponential phase of all PCR reactions. Data were analysed using the iCycle iQ system software (BioRad).

### Western blot analysis

Frozen tumour, normal urothelium tissue and cell lines were lysed in the PRO-PREPTM Protein Extraction Solution (iNtRON Biotechnology, Seoul, Korea) according to the manufacturer's instructions. Proteins were quantified by Bradford analysis. For western blot analysis, cell lysates were separated using 12% SDS–PAGE and then transferred onto nitrocellulose membranes (Millipore Corporation, Billerica, MA, USA). Membranes were blocked with 3% BSA in TBS-T buffer (150 mM NaCl, 10 mM Tris (pH 8.0) and 0.05% Tween 20) at room temperature for 1 h or overnight at 4°C, and then incubated with the following primary antibodies: ParvB (1 : 1000; Abnova, Newark, DE, USA), *β*-actin (1 : 50000, MAB1501; Chemicon, Temecula, CA, USA) or GAPDH (1 : 1000, MAB374; Chemicon); incubation period was 1 h at room temperature (ParvB) or overnight at 4°C (*β*-actin and GAPDH). Incubation with secondary antibodies (AP124P; Chemicon) was performed for 1 h at room temperature. Immunoblots were visualised using a chemiluminescence ECL system (Millipore Corporation), and western blot data were quantified using the BIO-PROFIL BIO-1D++ software.

### Stable knockdown of ParvB

HuSH 29-mer short-hairpin RNA (shRNA) constructs were generated against human ParvB (OriGene Technologies, Rockville, MD, USA). The shRNA (ATGTGGCTGAGGTGACACAGTCCGAAATA) construct was cloned in the pRS plasmid vector under the control of the U6 promoter. The control vector expressed shRNA against GFP shRNA. The different constructs were transfected into an urothelial cancer cell line (MGH-U1) at 60% confluency by using FuGENE reagent (Roche, Indianapolis, IN, USA) according to the manufacturer's instructions. After 72 h, the transfected cells were cultured under puromycin (Invitrogen) selection at 3 *μ*g ml^−1^ concentration. After 2 weeks of selected growth, several individual colonies were chosen and grown separately in a single well of a 12-well dish. Further selections of stably transfected cells were performed and grown in puromycin culture medium.

### DNA constructs

To generate expression vectors encoding the *TetR* gene, DNA fragments encoding the *TetR* gene was obtained from pcDNA6/TR expression vector (which includes the CMV promoter and rabbit *β*-globin intron II (IVS); Invitrogen) and then cloned into the *Pm*II*/Sa*II sites of the pRS expression vector (OriGene Technologies), resulting in the generation of a pRS-TetR expression vector. The 1.2-kb coding region of human *ParvB* gene was amplified from a pcDNA3.1myc/ParvB plasmid (kindly donated by Dr Hannigan) by PCR and then cloned into a pcDNA4/TO/VSVG vector to generate the pcDNA4/TO-ParvB-VSVG vector. All constructs were verified by nucleotide sequencing.

### Stable transfection and DOX-inducible overexpression of ParvB-VSVG

The pRS-TetR plasmid DNA was introduced into MCH-U1 cells using FuGENE transfection reagent (1 : 4). At 72 h post-transfection, the cells were subcultured into a 100-mm culture dish, and then subjected to puromycin at 3 *μ*g ml^−1^ concentration. MGH-U1 cell lines stably transfected with pRS-TetR in 24-well dishes were super-transfected with 0.3 *μ*g of pcDNA4/TO-ParvB-VSVG plasmid DNA. Again, after 72 h of transfection, the cells were subcultured into a 100-mm culture dish. In addition to puromycin, Zeocin (Invitrogen) at 300 *μ*g ml^−1^ concentration was added to cultures to select cell clones that were stably transfected with both pRS-TetR and pcDNA4/TO-ParvB-VSVG. We confirmed the presence of the stable clone overexpressing the *ParvB-VSVG* gene by stimulating the cells with 1 *μ*g ml^−1^ doxycycline (DOX). The stable clone with inducible ParvB-VSVG expression was designated ParvB-V2 (clone number 2).

### Cell growth curve assay

Cell lines were seeded on 12-well plates at a density of 5 × 10^4^ cells per well with complete culture medium and allowed to adhere to the plate overnight. For inducible cell lines, cells were cultured in triplicate with or without DOX (0.1 *μ*g ml^−1^) for 72 h at 37°C in a 5% CO_2_ atmosphere. The total number of cells that was counted in each plate was counted every 24 h and the mean value was calculated.

### Invasion assay

Invasion assays were performed in the transwell chambers with an 8-*μ*m pore size polycarbonate filter (Corning-Costar, Corning, NY, USA) coated with Matrigel (BD Biosciences, San Jose, CA, USA) for 1 h at 37°C in a serum-free medium. Cells were seeded on transwell inserts at a density of 5 × 10^4^ cells per well in the serum-free medium in the upper chamber (above the transwell insert) for 4 and 8 h at 37°C in a humidified 5% CO_2_ atmosphere. The lower chamber (below the insert) contained 10% foetal bovine serum, which was used as a chemoattractant. Following migration, the cells were fixed with methanol for 10 min and stained with Giemsa for 30 min. Cells were counted in three randomly chosen microscopic fields at 10 magnifications.

### Immunohistochemical staining

Immunohistochemistry (IHC) was performed, using the standard streptavidin–biotin complex method. Serial sections from appropriate pairs of normal urothelium and tumour tissue were cut and processed for IHC staining. Briefly, tissue sections (4 *μ*m thick) were deparaffinised with xylene and rehydrated in ethanol (Sigma Chemical Co., St Louis, MO, USA). For antigen retrieval, tissue sections were boiled in sodium citrate buffer for 20 min. Endogenous peroxidase activity was blocked by treatment with 3% hydrogen peroxide for 15 min and tissue sections were then incubated with 3% bovine serum for 30 min. Sections were incubated with primary monoclonal anti-ParvB antibody at 1 : 50 dilution overnight at 4°C. The sections were then further incubated with biotinylated goat anti-mouse antibody for 30 min followed by incubation with the streptavidin–biotin horseradish peroxidase complex for a further 60 min. The peroxidase reaction was visualised using a liquid DAB substrate kit (Dako, Carpinteria, CA, USA). All sections were counterstained with haematoxylin for 15 s. Sections incubated with phosphate-buffered saline were used as negative controls. Renal tubule tissues were used as positive controls. Two observers (W, CF and P, ST) blind to clinical features and pathology analysed the intensity of IHC staining using a consensus method. In brief, cytoplasmic IHC staining intensity in the cancer area was scored as absent, weak, medium or strong relative to adjacent normal renal tubule tissue or normal urothelium. The strong, medium and weak expressions in IHC were defined as positive staining and, in contrast, the absent expression as negative staining.

### Survival analysis and statistics

Disease-specific survival was determined using the Kaplan–Meier method, followed by the log-rank test. A univariate analysis with the log-rank test and Cox hazard regression was applied to assess the value of the following prognostic factors in predicting disease-specific survival: patient age (exceeding the mean age of the patient population), gender (male *vs* female), tumour grade (1 and 2 *vs* 3), tumour size (2, 2–5 or >5 cm), number of tumours (one focus *vs* multiple foci), tumour location (pelvis, ureter or both), ParvB staining (positive *vs* negative) and vascular invasion (determined microscopically). Statistical procedures were performed using commercially available statistical software (SPSS, SPSS Inc., Chicago, IL, USA).

## Results

### Aberrant expression of ParvB mRNA and protein in tumours and normal urothelium

**Figure 1:**
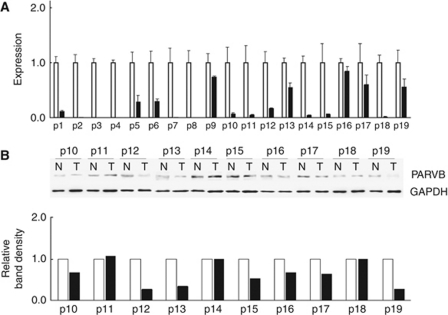
The study of ParvB mRNA (**A**) and protein level (**B**) in human upper urinary tract urothelial carcinoma in paired samples indicated that ParvB was downregulated in tumour sample as compared with normal tissue. Error bars indicate standard deviation for triplicate experiment data (□: normal urothelium; ▪: tumour).

We performed quantitative RT–PCR on RNA isolated from 19 patient-matched UUT-UC and normal urothelium tissues. Parvin-*β* mRNA expression was downregulated in all tumours (Figure 1A). As ParvB mRNA expression was downregulated in UUT-UC, we examined ParvB protein levels. Parvin-*β* protein levels from 10 patient-matched normal urothelium and tumour tissues were assessed. Downregulation of ParvB protein levels were detected in seven of 10 tumours (Figure 1B). Furthermore, ParvB protein levels were decreased by >50% in four of seven tumours. Our results show that ParvB is downregulated in UC tumours at both mRNA and protein levels.

### Dysregulation of ParvB influences cell growth and migration

**Figure 2:**
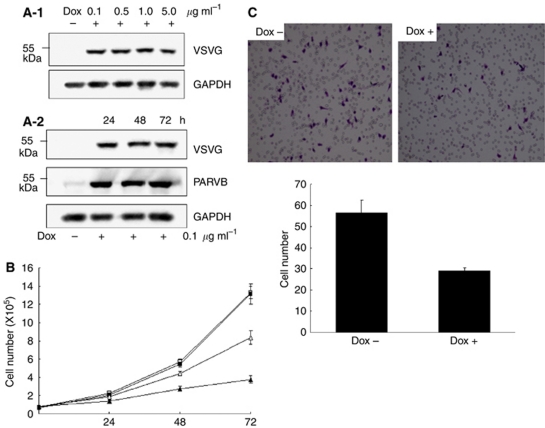
A DOX-inducible ParvB cell line was created and the expression of inducible ParvB is dependent on the concentration of doxycycline (**A**-**1**) and in a time-dependent pattern (**A**-**2**). (**B**) Overexpression of ParvB significantly decreased cell growth rate (*P*=0.012) and (**C**) inhibit the migration ability of the tumour cell as shown by the matrigel assay (*P*=0.001) (□: WT DOX− ▪: WT DOX+ ▵: Dox− ▴: Dox+)

**Figure 3:**
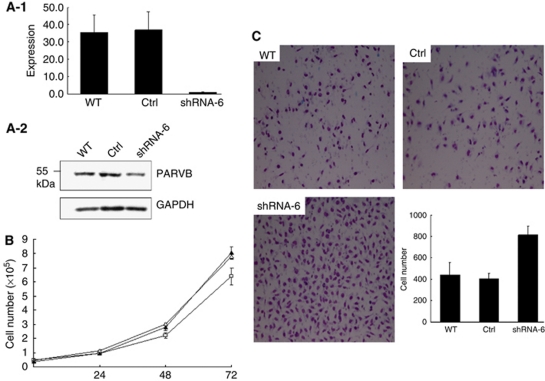
(**A**-**1** and -**2**) ParvB was successfully knocked down using siRNA in urothelial carcinoma cell line. (**B**) The knockdown of ParvB did not affect tumour cell growth rate, but (**C**) did significantly promote cell migration (*P*=0.000) (□: WT; △: Ctrl; ▴: shRNA-6).

The downregulation of ParvB expression in the UUT-UC tumours prompted us to investigate whether dysregulation of ParvB affects in cell growth and invasion. An MGH-U1-inducible cell line expressing the ParvB-VSVG protein, designated as ParvB-V2, was used for these experiments. We determined the minimum dose of DOX (0.1 *μ*g ml^−1^) that induced ParvB-VSVG protein expression (Figure 2A-1). The ParvB-V2 cell lines were treated with 0.1 *μ*g ml^−1^ of DOX for 24, 48 and 72 h. The expression of ParvB-VSVG protein was confirmed by western blotting (Figure 2A-2). Compared with the wild-type cell line, overexpression of ParvB-VSVG in the ParvB-V2 cell line resulted in significantly decreased cell growth (*P*=0.012) and migration (*P*=0.001) (Figure 2B and C). Thus, we proposed that downregulation of ParvB expression will increase cell growth and invasive properties. Parvin-*β*-siRNA-treated MGH-U1 cell lines were used to confirm this hypothesis. Downregulation of ParvB protein expression in ParvB-siRNA-treated MGH-U1 cell lines was confirmed by western blotting using a ParvB monoclonal antibody (Figure 3A). We also checked the ILK protein level, a ParvB interaction protein, which did not show significant change after the knockout of ParvB (data not shown). Compared with parental MGH-U1 cells transfected with vector only, reduced expression of ParvB in ParvB-siRNA-treated MGH U1 cells was accompanied by significantly increased cell migration (*P*=0.000) (Figure 3B and C). These results show that dysregulation of ParvB is associated with cell invasive properties in UC.

### Immunohistochemical staining and survival analysis

**Figure 4:**
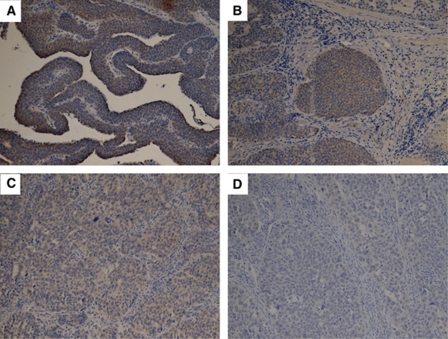
Examples of immunohistochemical staining intensity of ParvB in upper urinary tract urothelium cell carcinoma: (**A**) strong, (**B**) moderate, (**C**) weak and (**D**) absent.

**Figure 5:**
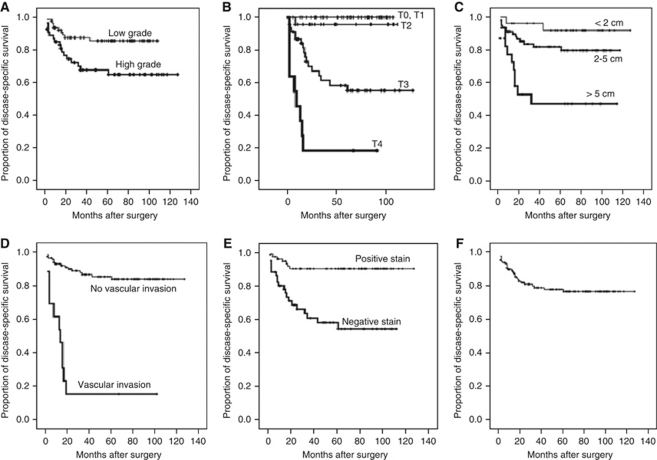
Kaplan–Meier estimation of disease-specific survival of patient with primary upper urinary tract urothelium cell carcinoma against: (**A**) tumour grade (*P*=0.0121); (**B**) pT stage (*P*=0.0000); (**C**) tumour size (*P*=0.0002); (**D**) tumour vascular invasion (*P*=0.0000); (**E**) ParvB staining intensity (*P*=0.0000); and (**F**) disease-specific 5-year survival curve.

To determine whether ParvB is a prognostic factor in patients with UUT-UC, immunohistochemical analysis of paraffin-embedded UUT-UC specimens was performed. The relationships between ParvB expression and clinical demographic characteristics of patients with UUT-UC are shown in Table 1. Immunohistochemistry revealed ParvB expression in the cytoplasm of UC cells. Comparison of ParvB expression levels revealed downregulation in UC specimens relative to normal urothelium, with the lowest level of ParvB expression in muscle invasive tumours relative to non-muscle invasive tumour (Figure 4). Moreover, this study also showed a strong negative correlation for staining intensity in relation to pT stage, tumour grade and tumour size (Table 1). In contrast, vascular invasive property, which significantly influences prognosis in patients with UUT-UC, did not correlate with ParvB expression (Table 1). We further investigated whether ParvB expression can be used as an independent prognostic factor. According to Kaplan–Meier analysis with the log-rank test, tumour grade, pT stage, tumour size, vascular invasion and downexpression of ParvB level were unfavourable factors for disease-specific survival of patients with UUT-UC (Figure 5). The disease-specific 5-year survival rate is 76%. Furthermore, Cox hazard regression analysis showed that pT stage, ParvB expression and vascular invasion were independent unfavourable factors for disease-free survival of patients with UUT-UC (Table 2).

## Discussion

ParvB is a focal adhesion binding protein highly expressed in the skeletal muscle, heart, spleen and kidney (Korenbaum *et al*, 2001). Parvin-*β*, also known as affixin, was identified first by Yamaji *et al* (2001). Parvin-*β* does not possess intrinsic catalytic activity and exerts its effect through interaction with other proteins such as ILK, *α*-actinin and *α*PIX (Sepulveda and Wu, 2006). The major protein that interacts with ParvB is ILK. Parvin-*β* contains 2 calponin homology (CH) domains, CH1 and CH2, in the C-terminal regions. Important functions have been identified for ParvB in integrin-mediated cell adhesion, regulation of cell morphology and cell motility, which are mediated through interactions with ILK by the CH2 domain. Although several ParvB isoforms have been identified, the biological functions of some of these proteins are unknown. However, because the CH domains show high conservation among various isoforms, they are potentially capable of interacting with ILK. Yamaji *et al* (2001) showed overexpression of the ParvB CH2 domain in CHO cell, which is phosphorylated by ILK inhibited cell migration. Furthermore, the interaction of ParvB with *α*-actinin is induced by the phosphorylation of ParvB CH2 domain, which is dependent on ILK kinase activity. Mishima *et al* (2004) found that interaction between ILK and ParvB is necessary for integrin signalling that results in Cdc42/Rac1 activation through *α*PIX. Zhang *et al* (2004) have shown that ParvA and ParvB are co-expressed in human cells. Parvin-*α* promotes intracellular ILK function, whereas ParvB has an inhibitory effect. Overexpression of ParvB inhibited the formation of the ILK–ParvA complex and promoted apoptosis in HeLa cells (Zhang *et al*, 2004). Mongroo *et al* (2004) showed that ParvB mRNA and protein levels are downregulated in breast cancer cell lines and advanced breast cancer tumours. These authors also showed that loss of ParvB expression leads to the upregulation of ILK activity in tumour cells, whereas expression of ParvB suppressed anchorage-dependent growth in breast cancer cell lines. Our analysis shows that ILK protein level did not alter after the downregulation of ParvB by siRNA. However, we would expect that ILK activity may be increased as shown by Mongroo in other cell line system. In addition, the same authors recently showed that ParvB suppressed breast cancer growth *in vivo* by decreasing cell proliferation (Johnstone *et al*, 2008). In this study, we found that ParvB mRNA and protein levels are downregulated in both UC cell lines and human UUT-UC cells. Moreover, we have shown that ParvB protein levels in advanced UUT-UC tumours are lower than those in superficial UUT-UC tumours. Similarly, IHC analyses performed in this study also revealed lower staining intensities for ParvB in cancer cells relative to normal urothelium cells. The extent of ParvB downregulation correlated with disease progression. We found that overexpression of ParvB in a DOX-inducible ParvB-V2 cell line resulted in suppressed cell growth and migration. In contrast, knockdown of ParvB expression in the MGH-U1 cell line enhanced cell invasive properties. However, lower levels of ParvB did not alter the growth rate of MCH-U1 cells. These results suggest that the expression of ParvB is essential for UC cell growth and migration.

Local tumour recurrence and metastasis are the major causes of mortality in patients with UUT-UC. Our results underscore ParvB as a regulator of cell growth and migration, which has important implications given that cell proliferation and invasiveness are related to tumour aggression. It remains unknown if ParvB expression levels in various cancers are related to patient survival. Mongroo *et al* (2004) first showed downregulation of ParvB expression in advanced breast cancer, although they did not examine if a correlation exists between ParvB expression and patients outcomes. To our knowledge, there are no previous reports with regard to the relationship between ParvB expression and cancer progression. This study is the first report that evaluated the possibility of a relationship between ParvB expression levels and clinical outcomes in UUT-UC patients and showed that ParvB expression is inversely correlated with the clinical progression of UUT-UC. This study showed that ParvB expression levels reversely correlated with tumour grade, tumour stage and tumour size. Furthermore, according to the univariate and Cox hazard regression analysis, high T stage, vascular invasion and low ParvB expression were unfavourable factors for disease-free survival. These clinical findings were matched to molecular results: low ParvB expression increased tumour cell growth and invasion.

Biological functions mediated by ParvB are dependent on interaction with ILK. ILK which is composed of N-terminal ankyrin repeats, a pleckstrin homology-like domain and a C-terminal kinase catalytic domain, is implicated in the regulation of anchorage-dependent cell growth, cell survival, cell motility, cell invasion and migration, angiogenesis and epithelial–mesenchymal transition (Hannigan *et al*, 2005). It is understood that ILK can activate a range of biological pathways depending on cell type in addition to being critical in development, which was shown by lethality in ILK-null mutant mice at the embryonic stage (Sakai *et al*, 2003). Recent evidence indicates that ILK expression is increased in various tumours, including prostate cancer, colon cancer, gastric cancer, ovarian cancer, Ewing's sarcoma, melanoma and breast cancer (Chung *et al*, 1998; Persad *et al*, 2000; Graff *et al*, 2001; Ito *et al*, 2003; Zellweger *et al*, 2005; Bravou *et al*, 2006; Rosano *et al*, 2006; Kim *et al*, 2007; Wong *et al*, 2007; Assi *et al*, 2008). To our knowledge, the role of ILK in UUT-UC has not been examined to date. This study showed the T-stage factor corresponding to the depth of tumour invasion; low ParvB expression corresponding to the ability of cell invasiveness and vascular invasion are the independent prognostic factors for disease-free survival. On the basis of our results, invasive capacity of tumour cells is an important factor related to prognosis in patients with UUT-UC. Interestingly, ParvB mediates its biological function by interacting with ILK; the signalling pathways induced by ILK may vary according to the cell type. The exact signalling pathways affected by knocking down ParvB expression in UUT-UC are not yet clear. In future study, it will be interesting to further dissect the role of ParvB-ILK and other potential signalling pathway that involve in UUT-UC pathogenesis.

## Figures and Tables

**Table 1:** Results of ParvB expression and clinicopathological characteristics in 129 patients with upper urinary tract urothelial cell carcinoma

		**Expression of ParvB**						
		**Positive**		**Negative**				
**Characteristics**	**No.**	***n***	**%**	***n***	**%**	***P*-value**	**Median PFS**	***P-*value for PFS**
Total no.	129	78	60.5	51	39.5			
*Gender*						0.542		0.306
Male	44	25	56.8	19	43.2		38	
Female	85	53	62.4	32	37.6		57	
*Age (years)*						0.155		0.255
<66.9	58	39	67.2	19	32.8		67	
>66.9	71	39	54.9	32	45.1		28	
*Location*						0.804		0.854
Pelvis	66	39	59.1	27	40.9		61	
Pelvis and ureter	26	15	57.7	11	42.3		10	
Ureter	37	24	64.9	13	35.1		55	
*Grade*						0.027		0.109
Low	76	52	68.4	24	31.6		56	
High	53	26	49.1	27	50.9		36	
*T stage*						0.000		0.000
Ta	31	28	90.3	3	9.7		61	
T1	18	17	94.4	1	5.6		83.5	
T2	23	9	39.1	14	60.9		48	
T3	46	20	43.5	26	56.5		17.5	
T4	11	3	27.3	8	72.7		3	
*Tumour size (cm)*						0.021		0.004
<2	27	21	77.8	6	22.2		67	
2–5	79	48	60.8	31	39.2		45	
>5	23	9	39.1	14	60.9		10	
*Tumour foci*						0.687		0.482
1	104	62	59.6	42	40.4		54.5	
≧2	25	16	64	9	36		40	
*Vessel invasion*						0.607		0.000
Positive	13	7	53.8	6	46.2		6	
Negative	116	71	61.2	45	38.8		58	

Abbreviations: ParvB=parvin-*β*; PFS=progression-free survival.

**Table 2:** Univariate and multivariate analysis of potential prognostic factors for disease-specific survival in patients with upper urinary tract urothelial cell carcinoma

	**Univariate**		**Multivariate**	
**Characteristics**	**Statistics**	***P*-value**	**95% CI**	***P*-value**
Gender	0.79	0.3739	0.364–1.979	0.705
Age	1.68	0.1953	0.847–6.412	0.101
Location	0.10	0.7494	0.859–3.649	0.150
Grade	6.30	0.0121	0.492–3.054	0.661
pT stage	41.57	0.0000	2.381–11.688	0.000
Tumour size	13.7	0.0002	0.380–1.872	0.844
Tumour foci	0.01	0.9210	0.402–3.661	0.731
Vascular	54.52	0.0000	1.465–8.785	0.005
ParvB staining	18.31	0.0000	0.161–0.987	0.047

Abbreviations: CI=confidence interval; ParvB=parvin-*β.*
